# NAFLD Fibrosis Progression and Type 2 Diabetes: The Hepatic–Metabolic Interplay

**DOI:** 10.3390/life14020272

**Published:** 2024-02-18

**Authors:** Simona Cernea

**Affiliations:** 1 Department M3, Internal Medicine I, George Emil Palade University of Medicine, Pharmacy, Science, and Technology of Târgu Mureş, 540142 Târgu Mureş, Romania; simona.cernea@umfst.ro or simonacernea@yahoo.com; 2 Diabetes, Nutrition and Metabolic Diseases Outpatient Unit, Emergency County Clinical Hospital, 540136 Târgu Mureş, Romania

**Keywords:** liver fibrosis, type 2 diabetes, NAFLD

## Abstract

The bidirectional relationship between type 2 diabetes and (non-alcoholic fatty liver disease) NAFLD is indicated by the higher prevalence and worse disease course of one condition in the presence of the other, but also by apparent beneficial effects observed in one, when the other is improved. This is partly explained by their belonging to a multisystemic disease that includes components of the metabolic syndrome and shared pathogenetic mechanisms. Throughout the progression of NAFLD to more advanced stages, complex systemic and local metabolic derangements are involved. During fibrogenesis, a significant metabolic reprogramming occurs in the hepatic stellate cells, hepatocytes, and immune cells, engaging carbohydrate and lipid pathways to support the high-energy-requiring processes. The natural history of NAFLD evolves in a variable and dynamic manner, probably due to the interaction of a variable number of modifiable (diet, physical exercise, microbiota composition, etc.) and non-modifiable (genetics, age, ethnicity, etc.) risk factors that may intervene concomitantly, or subsequently/intermittently in time. This may influence the risk (and rate) of fibrosis progression/regression. The recognition and control of the factors that determine a rapid progression of fibrosis (or its regression) are critical, as the fibrosis stages are associated with the risk of liver-related and all-cause mortality.

## 1. Introduction

Non-alcoholic fatty liver disease (NAFLD) has emerged as the most common chronic liver disease worldwide. It has been defined by the presence of steatosis in >5% of hepatocytes, as evidenced by histologic or specific imagistic methods (i.e., magnetic resonance imaging proton density fat fraction (MRI-PDFF) or magnetic resonance spectroscopy), in the absence of secondary causes of liver steatosis (such as excessive alcohol use, viral or autoimmune hepatitis, drugs, etc.) [1,2]. It comprises a spectrum of histopathological and clinical conditions, ranging from simple steatosis (±mild inflammation or ballooning, without evidence of hepatocyte injury) (non-alcoholic fatty liver, NAFL) to non-alcoholic steatohepatitis (NASH) (characterized by steatosis with inflammation and ballooning ± various degrees of fibrosis that can progress to liver cirrhosis) and hepatocellular carcinoma (HCC) [1,3].

The condition is viewed as part of a multisystemic disease, as it is frequently associated with components of the metabolic syndrome (type 2 diabetes mellitus (T2DM), obesity, and dyslipidemia) and cardio-renal comorbidities (cardiovascular disease, atrial fibrillation, chronic kidney disease, etc.) [4]. In fact, it has been considered the hepatic manifestation of the metabolic syndrome, with insulin resistance being a core pathogenetic mechanism [5,6]. In line with this, a panel of international experts proposed in 2020 a new terminology and definition criteria, which are more inclusive and homogenous than NAFLD [7]. The metabolic dysfunction-associated fatty liver disease (MAFLD) was defined as evidence of hepatic steatosis, associated with at least one of the following: overweight/obesity, T2DM, or metabolic dysregulation [7]. More recently, in June 2023, three scientific societies (the European Association for the Study of the Liver (EASL), the American Association for the Study of Liver Diseases (AASLD), and La Asociación Latinoamericana para el Estudio del Hígado (ALEH)) agreed upon the change in nomenclature to metabolic dysfunction-associated steatotic liver disease (MASLD) to reduce stigma and use affirmative criteria and definition instead, and the new nomenclature was endorsed by other professional organizations and societies [8,9]. MASLD was defined as hepatic steatosis with at least one of five cardiometabolic risk factors (in adults): body mass index (BMI) ≥ 25 kg/m^2^ or waist circumference ≥ 94/80 cm in Caucasian men/women (or ethnicity adjusted); fasting serum glucose ≥ 100 mg/dL, 2 h post-load glucose ≥ 140 mg/dL, glycated hemoglobin (HbA1c) ≥ 5.7%, T2DM, or treatment for T2DM; blood pressure ≥ 130/85 mmHg or specific drug treatment; plasma triglycerides ≥ 150 mg/dL or specific drug treatment; plasma high-density lipoprotein (HDL) cholesterol < 40 mg/dL for men and <50 mg/dL for women or specific drug treatment), in the absence of other causes [8]. Insulin resistance remains the key metabolic dysfunction underlying MASLD [8]. The new nomenclature is thought to bring a better understanding of the disease and increased awareness, as well as support for further biomarkers and drug development [8]. Thus, the changes refer not only to the terms, but also to the definition, since for MASLD the presence of a cardio-metabolic risk factor are required.

In this narrative review, we will discuss the factors associated with the presence and progression of NAFLD-related fibrosis, with special emphasis on the relationship with T2DM, and while transitioning to the new nomenclature, the terms NAFLD/NASH will still be used here.

## 2. NAFLD and T2DM Bidirectional Relationship

The significant burden of the disease is mirrored by its high global prevalence, as well as its hepatic and extrahepatic consequences. It is estimated that NAFLD affects about a quarter of the adult population worldwide, and slightly more than half of patients with T2DM, with some geographic differences [10,11,12]. More significant is the fact that the advanced stages of the disease are present in a larger proportion of patients with T2DM. Overall, the prevalence of NASH in the general population is about 2–6% worldwide, while in patients with T2DM it is 20–40% [10,11,12,13]. Similarly, the prevalence of advanced fibrosis (F2–4) was reported to be around 5–7% in the general population, and 12–20% in patients with T2DM [10,11,12,13,14,15,16]. Moreover, the risk of HCC is 2.0–2.5 fold higher in subjects with T2DM [17,18,19,20]. A recent individual participant-level data meta-analysis of six retrospective studies (2016 participants with NAFLD) indicated that T2DM was an independent predictor of incident HCC (adjusted hazard ratio (HR): 5.34 [1.67–17.09], *p* = 0.0048) and of incident hepatic decompensation (adjusted HR: 2.15 [1.39–3.34], *p* = 0.0006) [21].

Diabetes worsens the course of the disease, and in fact, histopathologic analyses of biopsy-proven NAFLD samples demonstrated that patients with T2DM have more advanced fibrosis [22,23,24]. There is a large body of literature that indicates T2DM as an important independent predictor and risk factor for advanced liver disease, including advanced fibrosis, cirrhosis, HCC, and liver-related hospital admissions and deaths [14,25,26,27,28,29,30]. The meta-analysis by Jarvis H et al. of 12 population-based cohort studies (22.8 million subjects followed-up for 10 years) has shown that T2DM doubles the risk of severe liver disease events (HR: 2.25 [1.83–2.76], *p* < 0.001) and increases the risk of fatal liver events by 63% [25]. The real-world study by Alexander M et al. of 18 million European patients has also demonstrated that diabetes predicts liver disease progression [31]. In NAFLD/NASH patients, the strongest association with liver outcomes (cirrhosis or HCC) is seen in patients with diabetes at baseline (HR: 2.3 [1.9–2.78]) [31]. Similar results were reported in the study conducted by Kanwal et al. that included 271,906 patients with NAFLD which were followed-up for 9.3 years [26]. Diabetes was the only metabolic risk factor independently associated with the risk of progression to HCC (adjusted HR: 2.77 [2.03–3.77]), and it also increased the risk of cirrhosis by 89% [26].

This bidirectional relationship between NAFLD and T2DM is further substantiated by the results of the retrospective analysis performed by Colosimo and colleagues (637 patients with T2DM) that evaluated the impact of optimized glycemic control on markers of liver steatosis and fibrosis [32]. The change in HbA1c corelated positively with changes in Fibrosis-4 (FIB-4) (after adjustment for confounders) (R = 0.666, overall model test *p* = 0.001; ANOVA for change in HbA1c, *p* = 0.037) and with Fatty liver index (FLI) independent of body mass index (BMI) change [32].

It is apparent though that not all persons with diabetes have the same risk of hepatic steatosis and fibrosis, and this is modulated by insulin resistance. Diabetes is a heterogenous disease and in recent years several novel subtypes have been described by cluster analysis [33]. Apparently, these subtypes have different associations with liver outcomes [33,34,35]. Severe insulin-resistant diabetes (SIRD), characterized by insulin resistance (high homoeostasis model assessment 2-insulin resistance (HOMA2-IR) index) and obesity (high BMI), had the highest prevalence of NAFLD (24.1%) in the study by Ahlqvist et al. [33]. The NAFLD-associated rs10401969 variant of the Transmembrane 6 Superfamily Member 2 (*TM6SF2*) gene was also associated with SIRD [33]. Zaharia et al., who analyzed data from 1105 participants in the German Diabetes Study with newly diagnosed diabetes, also reported that patients assigned to the SIRD cluster had the highest hepatic lipid content and FLI, as well as the highest estimates of liver fibrosis (NAFLD fibrosis score and aspartate aminotransferase (AST)-to-platelet ratio index (APRI)), and after 5 years of follow-up, the prevalence of hepatic fibrosis remained higher in this cluster [34]. Moreover, the same group showed that SIRD had a higher prevalence of the rs738409 (G) polymorphism of the patatin-like phospholipase domain-containing 3 (*PNPLA3*) gene, which is associated with increased risk and progression of NAFLD [35].

On the other hand, there are convincing data in the literature coming from several meta-analyses which indicate that NAFLD increases the risk of diabetes by about two-fold [36,37,38]. Importantly, the meta-analysis by Mantovani et al. (33 studies, 501,022 individuals, median follow-up of 5 years) has also indicated that the severity of hepatic fibrosis parallels the risk of incident diabetes, independent of other risk factors such as age, sex, adiposity, or metabolic comorbidities [35]. This was confirmed by several longitudinal biopsy studies showing higher risk of incident T2DM in NAFLD patients with significant (≥F2) or progressive fibrosis [39,40,41].

It appears that the inverse relationship is also valid in the sense that improvement or resolution of NAFLD reduces the risk of T2DM and even favors its remission [42,43,44,45]. The retrospective cohort study by Yamazaki et al. (3074 ultrasonography (US)-evaluated participants followed-up for >10 years) reported a reduced incidence of T2DM by about 70% (multivariate odds ratio (OR): 0.27 [0.12–0.61]) with US-defined NAFLD improvement [42]. The same group later reported that improvement in US-detected hepatic steatosis was associated with a higher chance of T2DM remission (adjusted OR: 3.08 [1.94–4.88]) over a follow-up period of about 2 years [44]. A Chinese prospective cohort study evaluated data from 5671 participants and also suggested a favorable effect of US-defined NAFLD improvement on the risk of progression to new onset T2DM (OR: 0.50 [0.32–0.80]), and on diabetes remission (OR: 2.06 [0.96–4.42]) [45].

Thus, T2DM doubles the risk of NAFLD and significantly increases the risk of progression toward the advanced stages of the disease, while NAFLD doubles the risk of diabetes, mainly when advanced fibrosis is present. Improvement in one condition seems to bring favorable effects to the other, but more data are still needed to further validate these findings.

## 3. Natural History of NAFLD

The classic view on the natural history of NAFLD is that hepatic steatosis is rather a benign condition with a slow progression, while NASH has in fact the potential to advance to fibrosis and cirrhosis [46]. This dichotomous perspective is challenged by newer histological studies, and it is apparent that the natural course of the disease is rather heterogenous, variable, and dynamic, with some NAFL patients progressing toward more advanced stages of fibrosis, some patients being “slow”, while others “fast” progressors, and some patients presenting regression of fibrosis, even from more advanced stages [3,46,47,48].

A meta-analysis of histological studies (11 studies; 411 patients with paired liver biopsies collected at least one year apart) indicated that hepatic fibrosis progressed in 33.6% of patients, remained stable in 43.1%, while 22.3% of cases presented an improvement in fibrosis stage [49]. The annual fibrosis progression rate was estimated to be 0.14 [0.07–0.21] stages in patients with NASH at baseline (corresponding to one stage progression over 7.1 years), and 0.07 stages [0.02–0.11] in those with steatosis alone (corresponding to one stage of progression over 14.3 years) [49]. A more recent meta-analysis by Pe L et al. that included 54 studies (observational and randomized controlled trials (RCTs), with 26,738 patients with NAFLD diagnosed by liver biopsy or imaging) reported that rates of fibrosis progression were similar between baseline fibrosis stages, and the time to progress by one stage varied between 9.9 and 22.2 years from F0 to F3 [50]. In the same study, it was shown that hepatic steatosis resolution occurred in 21% of subjects with NAFL at baseline after about 4.5 years, while 29% of NASH patients presented resolution of steatohepatitis after a median of 3.5 years [50].

Several meta-analyses of histological data from the placebo arms of the RCTs have evaluated the changes in NASH severity over time and thus provided a valuable insight into the natural history of the disease. The study by Ng CH et al. included 43 RCTs (2649 placebo-treated NASH patients) and showed spontaneous improvements in liver histology over time: the pooled estimate of NASH resolution was 11.65% [7.98–16.71], the two-point NAFLD activity score reduction without worsening of fibrosis was 21.11% [17.24–25.57], and the rate of at least one stage reduction in fibrosis was 18.82% [15.65–22.47] [51]. About 23% of patients presented fibrosis progression of ≥ 1 stage [51]. The network meta-analysis conducted by Penissi et al., which evaluated data from 15 phase 2 and 3 RCTs, reported a pooled estimate rate of NASH resolution (without worsening of fibrosis) of 17% ([12–23%], *p* < 0.01), and of 21% ([13–31%], *p* < 0.01) for at least one stage of fibrosis improvement without worsening of NASH [52]. Finally, Ampuero and colleagues reported in their meta-analysis of 27 RCTs a pooled efficacy for NASH resolution of 10% [7,8,9,10,11,12], and 18% [15,16,17,18,19,20,21] for improvement in fibrosis in the placebo arms [53].

However, the evolution of NAFLD/NASH in patients with diabetes is insufficiently investigated so far. A nation-wide cohort study from Italy that included 5030 patients with T2DM evaluated hepatic steatosis using the FLI over a period of 3 years and indicated that about 5% of T2DM patients develop and have remission of liver steatosis each year, respectively, suggesting a dynamic evolution in this patient category as well [54]. More recently, a large multicenter study of 3446 paired-liver biopsied patients with NAFLD evaluated the incidence of fibrosis progression in individuals with or without T2DM [55]. More patients with NAFLD and T2DM presented progression of fibrosis (from stages F0–2 to F3–4) compared to NAFLD subjects without T2DM (26.0% vs. 14.1%, *p* = 0.008), and similar proportions of patients presented fibrosis regression (from F3–4 to F0–2) in the two groups (27% vs. 22%, *p* = 0.52) [55]. The adjusted rate of fibrosis progression rate was higher in NAFLD patients with T2DM (+0.23 [0.39] versus +0.16 [0.26] stage/year, *p* = 0.048), who also had a higher adjusted cumulative incidence of fibrosis progression by at least one stage (24% vs. 20% at 4 years, 60% vs. 50% at 8 years, and 93% vs. 76% after 12 years) [55]. The presence of T2DM at baseline, but not HbA1c, was an independent and significant predictor of fibrosis progression (adjusted HR: 1.69 [1.17–2.43], *p* = 0.005) [55]. However, the incidence of fibrosis regression by ≥1 stage was similar between the two groups, and T2DM did not emerge as a predictor of fibrosis regression [55]. A Japanese cross-sectional multicenter study (1365 biopsy-proven NAFLD) identified diabetes as a significant risk factor for advanced fibrosis (F3–4) (multivariate OR: 2.387 [1.603–3.553], *p* < 0.0001) [56].

Thus, the natural history of NAFLD in patients with and without T2DM does not evolve in a straightforward and predictable manner, and this is most probably driven by the interaction of a variable number of risk factors and the complex pathophysiological mechanisms involved.

## 4. Factors Associated with Fibrosis Progression and Regression

It is apparent that the disease trajectory is highly variable due to interference of a number of multilayered risk factors and therapies that may critically influence disease progression and regression. Identifying the factors associated with fibrosis progression, especially rapid progression toward the advanced stages, and also those associated with fibrosis regression is important, as this might enable improved monitoring strategies and support biomarkers and drug development. This is relevant also because fibrosis stage was proven to be associated with the risk of mortality and is the most significant predictor of outcomes [57,58]. A retrospective study with a mean follow-up period of 20 years (646 biopsy-proven NAFLD patients) had previously shown that fibrosis stage and not NASH increased the risk of mortality (HR: 3.04 [1.94–4.78], *p* < 0.001 for F3 vs. F0, and 6.53 [3.55–12.03], *p* < 0.001 for F4 vs. F0) [57]. This was also demonstrated in the meta-analysis by Dulai et al. (five cohort studies; 1495 NAFLD patients, with 17,452 patient years of follow-up) [58]. Compared to F0, higher fibrosis stages exponentially increased the risk of liver-related mortality (rate ratios: 1.41 [0.17–11.95], 9.57 [1.67–54.93], 16.69 [2.92–95.36], and 42.30 [3.51–510.34] from F1 to F4), respectively, and also of all-cause mortality (rate ratios: 1.58 [1.19–2.11], 2.52 [1.85–3.42], 3.48 [2.51–4.83], and 6.40 [4.11–9.95] from F1 to F4) [58]. In addition, the presence of advanced liver fibrosis modifies the clinical management, as it requires periodic surveillance for HCC, and in patients with T2DM it may influence the choices of antihyperglycemic therapeutic agents (mainly in advanced cirrhosis, when insulin might become the only option) [17,59].

**Figure 1:**
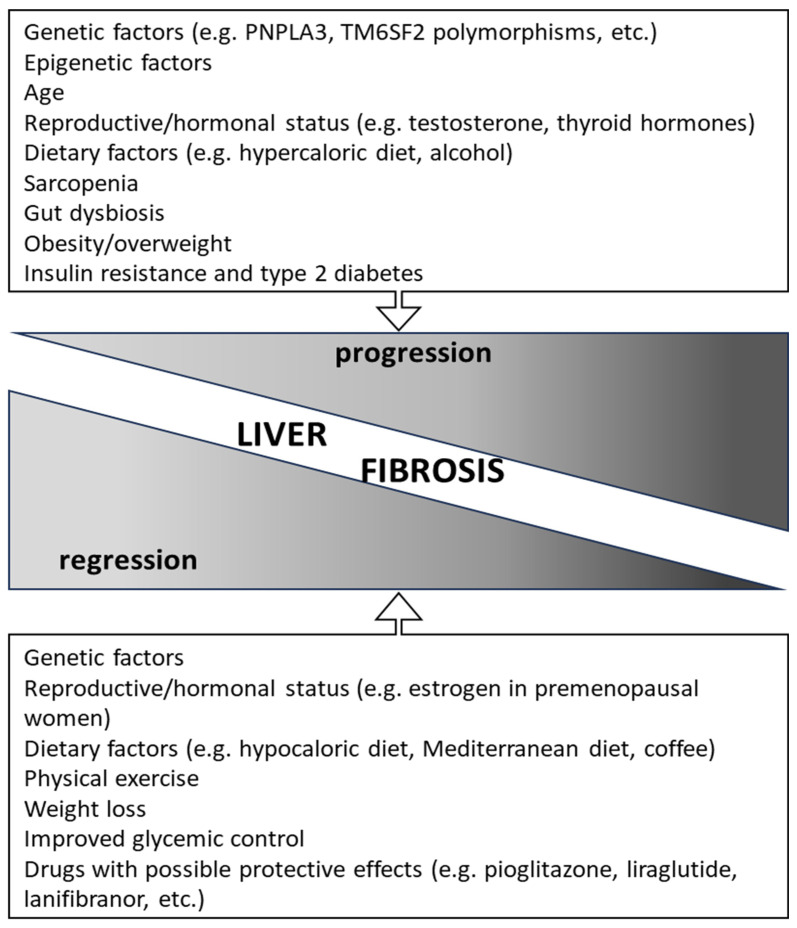
Factors associated with the progression/regression of NAFLD-associated liver fibrosis (see text for details).

A number of risk factors for disease progression have been described (Figure 1). Some of them are modifiable (e.g., diet, physical exercise, microbiota composition, etc.) and others are not (e.g., genetics, age, ethnicity, etc.), and these may act concomitantly, in a synergistic manner, or subsequently/intermittently through the lifetime of NAFLD patients, and thus may influence the risk (and rate) of fibrosis progression.

Genetic and epigenetic factors. The genome-wide association studies (GWAS) have identified several gene loci associated with the risk of NAFLD development and progression [60].

The polymorphism of *PNPLA3*, which codes adiponutrin, a protein involved in lipid remodeling of hepatic triglycerides, mediates NAFLD risk: the single-nucleotide polymorphism (SNP) rs738409 determines a missense variation (I148M) that disrupts the enzymatic activity interfering with lipid catabolism, and has been shown to associate with liver fibrosis and disease progression [61,62,63]. The *PNPLA3* I148M also alters retinol release from the hepatic stellate cells (HSCs), determining a subsequent reduction in the secretion of matrix-modulating enzymes and changing the extracellular matrix remodeling, and this might explain the association with hepatic fibrosis development and progression [60,64]. The meta-analysis by Singal et al. (24 studies with 9915 patients) reported that the *PNPLA3* rs738409 SNP is associated with fibrosis severity (OR: 1.32 [1.20–1.45]), and the risk was similar in patients with NAFLD and with other liver disease etiologies [63].

However, the gene–diet interaction seems to be an important factor that modulates the effect of the SNP on the risk of fibrosis [63]. In a study that included 452 non-Hispanic white NAFLD subjects, higher carbohydrate intake was positively associated with higher risk of significant fibrosis (≥F2) (adjusted OR: 1.03, *p* < 0.01), while higher intakes of n-3 polyunsaturated fatty acids (n-3 PUFAs), methionine, choline, and isoflavones presented an inverse association (adjusted OR of 0.17, 0.32, 0.32, with *p* < 0.01, and 0.74, *p* = 0.049, respectively) [65]. The *PNPLA3* rs738409 G-allele significantly modulated the relationship between the intakes of the above-mentioned dietary components and fibrosis severity in a dose-dependent manner [65]. Moreover, a recent analysis of data from two independent cohorts (7893 and 46,880 participants, respectively) indicated that the *PNPLA3*-rs738409-GG had additive effects with metabolic risk factors, such as diabetes and obesity: carriers of this genotype with an indeterminate risk of fibrosis (FIB-4 score between 1.3 and 2.67) and diabetes had a similar incidence rate of cirrhosis as patients with a high risk of fibrosis (FIB-4 > 2.67) [66].

The GWAS also identified the *TM6SF2* variant rs58542926 as being associated with NAFLD [67]. *TM6SF2* is a transmembrane protein involved in the secretion of VLDL cholesterol, and subsequent studies have associated this SNP with a higher risk of advanced fibrosis in patients with NAFLD, independent of other risk factors [60,68,69]. This was not confirmed in the study by Krawczyk et al., which in turn showed that the membrane-bound O-acyltransferase domain containing 7 (*MBOAT7*) rs641738 variant was associated with the development of hepatic fibrosis (OR: 1.446, *p* = 0.046) [70]. A meta-analysis of 42 studies which analyzed data from 1,066,175 participants (9688 with liver biopsies) demonstrated that the rs641738 variant was positively associated with advanced fibrosis in Caucasian adults (OR: 1.22 [1.03–1.45], *p* = 0.021) [71]. Variants of other genes, such as glucokinase regulatory protein (*GCKR*), which regulates glucose metabolism and de novo lipogenesis, have been correlated with NAFLD-associated fibrosis [72]. The *GCKR* rs780094 SNP was independently linked to the severity of liver fibrosis (OR: 2.06 [1.43–2.98], *p* < 0.001) in an Italian histologic study (366 NAFLD patients) [73].

On the other hand, several gene polymorphisms appear to confer protection from hepatic fibrosis (e.g., Krueppel-like factor (*KLF*) *6* rs3750861, interleukin (*IL*) *28B* rs12979860, Superoxide dismutase (*SOD*) *2* rs4880, *MER protocol-oncogene*, tyrosine kinase (*MERTK*) rs4374383, etc.) [74]. A high-throughput RNA sequencing approach analyzing 206 samples from a histologically defined NAFLD cohort revealed and validated a gene signature for fibrosis progression, with 25 genes differentially expressed through fibrosis stages F2 to F4, and further analysis at the protein level identified aldo-keto reductase family 1 member B10 (AKR1B10) and growth/differentiation factor 15 (GDF15) concentrations as being strongly correlated with fibrosis stage [75].

A retrospective cohort study from southern Italy including 454 participants with NAFLD and with or without T2DM found no significant differences in the distribution of SNPs between the two patient categories [76]. Patients with T2DM who carried the risk alleles had a higher risk of liver fibrosis and significantly higher liver stiffness [76]. Moreover, a recent longitudinal study (407 T2DM-MASLD patients followed-up for 11 years) showed that combined polymorphisms of the *PNPLA3* and *TM6SF2* (two or more risk alleles) significantly increased the risk of cirrhosis (OR: 18.48 [6.15–55.58]; *p* < 0.001) and of cirrhosis complications (OR: 27.20 [5.26–140.62]; *p* < 0.001) [77]. The same impact of the combination of the risk alleles (common genetic variants of *PNPLA3*, *TM6SF2*, and *HSD17B13*) on the severity of NAFLD was also demonstrated by a large cohort study (110,761 individuals from Denmark and 334,691 individuals from the UK Biobank) [78]. A higher number of risk alleles (higher genetic risk score) progressively increased the risk of cirrhosis (up to 12-fold higher) and of HCC (up to 29-fold higher) in individuals from the general population [78].

Epigenetic modifications have also been shown to modulate liver fibrogenesis and they might in fact explain the variability in disease progression and regression in NAFLD patients with similar gene polymorphisms [74,79]. They have been suggested as potential non-invasive markers of disease progression [60]. A differential DNA methylation of pro- or antifibrogenic genes (i.e., transforming growth factor-β (*TGFβ*)*1*, platelet-derived growth factor (*PDGF*)*α*, or peroxisome proliferator-activated receptor (*PPAR*)*α* and *PPARδ*), genes encoding matrix molecules or remodeling factors, but also chemokines (CCR7 and CCL5) or factors related to the inflammasome (e.g., signal transducer and activator of transcription (STAT)1, and caspase 1 (CASP1)) has been observed in patients with NAFLD, and this was correlated to the fibrosis stage [80,81,82]. In addition, some emerging data suggest that a dysregulated microRNA (miRNA) expression pattern occurs in NAFLD and may influence disease progression/fibrogenesis (e.g., downregulation of miR-122, miR-331-3p, and miR-30c) [74,83,84]. Also, there are sparce data regarding the role of long non-coding RNAs (lncRNAs) in humans with NAFLD fibrosis [85]. Several lncRNAs (such as nuclear enriched abundant transcript 1 (NEAT1), metastasis-associated lung adenocarcinoma transcript 1 (MALAT1), and plasmacytoma variant translocation 1 (PVT1)) proved to be differentially expressed in fibrotic samples from NAFLD patients [85]. Nevertheless, data connecting the ncRNAs and NAFLD-associated fibrosis are not robust enough, and more studies are still needed in this direction [74].

Ethnicity/race. Data in the literature seem to indicate ethnic disparities related to the prevalence of NAFLD/NASH, but this might be influenced also by genetic or socio-economic factors, lifestyle habits, or other factors [86]. The meta-analysis by Rich et al. of 34 studies (368,569 patients) showed that Hispanics had the highest prevalence of NAFLD and NASH, Blacks had the lowest NAFLD/NASH prevalence, while the percentages were intermediate in Whites [87]. Nevertheless, the prevalence of significant fibrosis was similar among the ethnic/racial groups [87]. NAFLD prevalence seems to be similar in Asian Americans as compared to non-Asian Americans, despite the lower prevalence of obesity in this racial group, while the fibrosis stages were similar, as indicated by data from the National Health and Nutrition Examination Surveys 2017–2018 [88].

Age. Some (but not all) studies observed an inverse U-shaped relationship between age and NAFLD prevalence [89]. However, the meta-analysis by Younossi et al. reported a global NAFLD prevalence consistently increasing throughout age categories [10]. Aging also seems to be associated with more severe liver fibrosis [90,91].

Sex, reproductive, and hormonal status. There are sex-based differences in NAFLD/NASH prevalence and severity, partly related to sex hormones [92]. A recent systematic review and meta-analysis of 54 studies (62,239 for NAFLD analysis, and 6444 for the advanced fibrosis analysis) indicated a lower risk of NAFLD in women compared to men, but a higher risk of advanced fibrosis in those with established NAFLD (mainly after 50 years of age) [93]. In premenopausal women, estrogen seems to exert a protective effect against hepatic steatosis and fibrosis, while high testosterone levels double the risk of NASH fibrosis [92,94]. In a cross-sectional study that included 1782 male subjects with T2DM, total testosterone levels were associated with fibrosis progression (adjusted OR: 0.45 [0.29–0.72], for Q1 vs. Q4, *p*_trend_ = 0.001) [95]. In addition, a number of dysregulations of endocrine axes (hypothyroidism, growth hormone deficiency, hypercortisolemia, etc.) are associated with NAFLD/fibrosis development and progression [96,97]. There are some suggestions that thyroid hormone levels are associated with the risk of progressive hepatic fibrosis in patients with T2DM, NAFLD, and normal thyroid function [98].

Diet and alcohol intake. There are sparce high-quality data evaluating the impact of diet or dietary components on liver fibrosis progression in subjects with NAFLD/NASH [17]. Higher caloric intake was shown to be positively associated with NAFLD, but there is not enough solid evidence regarding the correlations with the progression of NASH-associated fibrosis [99]. Nevertheless, a hypocaloric diet (with or without physical activity) leading to weight loss (of >10%) has been shown to improve liver fibrosis [100].

Data from several observational studies indicated that adherence to the Mediterranean diet was associated with a lower risk of hepatic fibrosis [101,102,103]. The Med-Diet score appeared to be negatively correlated with markers of liver fibrosis (N-terminal pro-peptide of type III collagen (PRO-C3)) [104]. Moreover, an intervention study in 144 subjects with moderate or severe NAFLD demonstrated that a low-glycemic-index Mediterranean diet alone or combined with a physical activity program (aerobic with/without resistance exercises) was associated with significant reduction in liver fibrosis (estimated by vibration-controlled elastography) after 90 days [105]. Similar results were shown by a previous smaller study [106]. In addition, a study that included 170 subjects with NAFLD reported that adherence to a healthy dietary pattern (characterized by a high intake of nuts, vegetables, fruits, vegetable oils, low-fat dairy, white meat, coffee, and tea) was associated with a lower risk of liver fibrosis (OR: 0.26 [0.10–0.49]), while the Western dietary pattern (defined by a high intake of refined grains, meat, potatoes, eggs, and soft drinks) was associated with higher risk (OR: 4.21 [1.63–8.31]) [107]. A higher intake of hydrogenated fats, read meat, and soft drinks was associated with an increased risk of fibrosis [107]. Similarly, analysis of data from the Framingham Heart Study and the National Health and Nutrition Examination Survey indicated inverse associations between higher diet quality and liver fibrosis [108]. A small RCT (44 subjects with NAFLD) also demonstrated that adherence to a modified alternate-day calorie restriction diet for eight weeks resulted in a significant decrease in liver fibrosis (SWE score: −0.74 [0.19–1.29], *p* = 0.01) compared to a habitual diet [109].

Several dietary components have been associated with different risks of hepatic fibrosis in NAFLD patients. For example, dietary intake of myristic acid appeared to be higher in patients with NAFLD fibrosis (*p* = 0.02), while deficient choline intake was associated with higher liver fibrosis in post-menopausal women with NAFLD (*p* = 0.002) [110,111]. On the other hand, a meta-analysis of eleven studies demonstrated a protective effect of coffee consumption against liver fibrosis (RR: 0.65 [0.54–0.78], *p* < 0.00001) [112]. A small 12-week intervention study showed that a low free-sugar diet may reduce hepatic fibrosis in NAFLD subjects with overweight/obesity [113].

Although some cross-sectional studies have suggested that moderate alcohol intake is associated with less severe fibrosis in NAFLD patients, others have shown opposite results, while several longitudinal studies have demonstrated that even a modest alcohol consumption (>10 g/day) or heavy binge drinking may worsen fibrosis and increase the risk of liver-related outcomes [114,115,116,117].

Physical activity and sarcopenia. Several studies have indicated an independent association between NAFLD-related fibrosis and sarcopenia [118,119,120,121]. There are some indications that sarcopenia and/or severe myosteatosis are also associated with fibrosis progression, but more data are needed to substantiate these findings [122,123]. Myokines are suggested to be the mediators of the altered liver–muscle crosstalk, but sarcopenia and NAFLD fibrosis also share some other pathophysiological pathways (i.e., insulin resistance, chronic inflammation, alterations in the regulation of hormone signaling, etc.) that might explain the bidirectional link between them [124,125]. In fact, exercise-induced irisin might mediate the effect of physical activity on NAFLD through its anti-inflammatory and antioxidant properties [126,127]. Indeed, some but not all studies indicated a reduction in liver fibrosis with high-intensity interval training or moderate-to-vigorous aerobic exercise [128,129,130]. Additional research is needed, however, in order to decipher the independent effect of exercise on biopsy-proven NAFLD fibrosis.

Gut microbiota. The altered gut–liver axis has been linked to NAFLD pathogenesis through a number of mechanisms that include the dysbiosis of gut microbiota, which favors disease progression by increasing inflammation, hepatocellular toxicity/damage, and fibrogenesis [131,132]. Gut dysbiosis increases intestinal permeability, favoring toxic bacterial product translocation, higher free fatty acid (FFA) absorption, increased pro-inflammatory cytokines, lipopolysaccharide and ammonia, also intestinal inflammation and dysmotility, etc. [131,133]. Patients with NAFLD have an altered composition of gut microbiota and exhibit a distinct microbial signature, although the microbiome profile is not entirely deciphered [134]. It seems though that there is an increase in the Bacteroidetes and a decrease in the Firmicutes phyla associated with significant liver fibrosis, although other changes have been observed (increase in Proteobacteria phylum and several species such as Bacteroides, Escherichia coli, and possibly Ruminococcus; decrease in Prevotella, etc.) [135,136,137]. Modulation of the gut microbiota through lifestyle intervention might influence the disease/fibrosis progression, as suggested by the subanalysis of the PREDIMED-Plus trial data, but more evidence is needed in this respect [138].

Obesity. Excessive (and dysfunctional) body adiposity, mainly abdominal/visceral obesity, has been linked to the development and progression of NAFLD/liver fibrosis through the release of pro-inflammatory cytokines, FFAs, and adipokines, and hepatic insulin resistance [139,140,141]. Two large cohort studies from Korea (59,957 and 40,700 adults with NAFLD, respectively) showed that obesity/weight gain correlated with worsening of hepatic fibrosis (assessed by non-invasive biomarkers) over a median follow-up period of 7.7 and 6.0 years, respectively [142,143]. Another longitudinal cohort study from Spain (1478 adult subjects) showed that abdominal obesity was associated with moderate-to-advanced liver fibrosis development over a median follow-up of 4.2 years (β: 0.27 [0.11–0.43], *p* = 0.001) [144]. On the other hand, intervention studies have demonstrated that significant weight loss (≥10% body weight) through lifestyle intervention was associated with the regression of fibrosis in a significant proportion (45%) of NAFLD patients [100]. The analysis of data from two RCTs also demonstrated that for each kg of weight loss, there was a 5% ([2–8%], *p* = 0.001) increase in the odds of fibrosis improvement [145].

Insulin resistance and type 2 diabetes. As mentioned above, the evidence from the literature points to T2DM as an important metabolic condition associated with the progression of NAFLD-related fibrosis, as well as with a more advanced fibrosis. By using a non-invasive marker of fibrosis (FIB-4) in a sample population of 266 subjects with both NAFLD and T2DM, we have found that about half of them had a significant risk of fibrosis (FIB-4 ≥ 1.3), and 9.4% presented a high risk of advanced fibrosis (FIB-4 ≥ 2.67) (Cernea S., data not published). Moreover, longitudinal studies indicate overall that T2DM is associated with a higher risk of progression of fibrosis [146,147,148]. In addition, a recent case–control study (161 T2DM offsprings and 78 controls) has shown that parental history of T2DM increased the risk of NAFLD-associated significant fibrosis evaluated by transient elastography (OR: 8.89 [1.09–72.01], *p* = 0.041) after adjustment for confounding factors (age, gender, metabolic traits, and *PNPLA3* and *TM6SF2* polymorphisms) [149]. Previous research has indicated that parental history of T2DM is associated with a higher risk of NAFLD and higher non-fasting liver enzymes, with body adiposity being a substantial contributing factor [150,151].

Apparently, early glucose derangements (prediabetes, higher glucose variability as evidenced by the continuous glucose monitoring system) and higher post-prandial blood glucose are also correlated with the severity of liver fibrosis, suggesting that hyperglycemia per se might be involved in fibrosis progression [144,152,153,154]. In patients with NAFLD and T2DM, the increase in HbA1c was significantly associated with liver fibrosis progression (standardized coefficient: 0.17 [0.009–0.326], *p* = 0.038) in a liver biopsy study [155]. The mechanisms behind these associations are not entirely clear, but a gene expression analysis suggested that diabetes may induce hypoxia and oxidative stress in hepatocytes, which may mediate inflammation and fibrosis [155]. In fact, hyperglycemia/glucotoxicity and lipotoxicity induce oxidative stress, mitochondrial dysfunction, and endoplasmic reticulum stress that are involved in the occurrence and progression of NAFLD [156]. Gluco- and lipotoxicity are interrelated and further aggravate insulin resistance [157]. Insulin resistance is, at least in part, the link between T2DM and NAFLD, is involved in the pathogenesis of both conditions, and is a key contributor to NAFLD progression [48]. Moreover, other research indicated that high fasting insulin concentrations or treatment with insulin increased the risk of progression to advanced fibrosis (OR: 1.36, *p* < 0.001) [158,159]. It has been suggested that insulin is pro-fibrinogenic, as it induces the proliferation of HSCs, the upregulation of connective tissue growth factor, and collagen synthesis [160,161].

On the other hand, antihyperglycemic agents seem to bring benefit in NAFLD, including fibrosis, although more RCTs in biopsy-proven NAFLD patients are still needed for some of the agents. Incretin-based therapies were associated with a decrease in serum alanine aminotransferase (ALT) (−14.1 IU/L [8.3–19.8], *p* < 0.0001), as indicated by a meta-analysis of four studies (136 participants with NAFLD), with two of them indicating a reduction in liver fibrosis [162]. Two RCTs have shown that treatment with glucagon-like peptide-1 (GLP-1) receptor agonists reduced the progression of hepatic fibrosis [163,164]. A meta-analysis of eight RCTs employing thiazolidinediones in biopsy-proven NASH patients demonstrated that pioglitazone was associated with improvement in fibrosis of any stage (OR: 1.77 [1.15–2.72], *p* = 0.009), including advanced fibrosis (OR: 4.53 [1.52–13.52], *p* = 0.007) [165]. In addition, some preliminary results also suggested potential benefits of the sodium–glucose cotransporter 2 (SGLT 2) inhibitors on the markers of liver fibrosis [166,167].

Transaminases levels. Some studies have found that a higher ALT level or a low AST/ALT ratio are associated with significant fibrosis (or development of progressive fibrosis), but ALT is rather a minor independent factor that contributes to identifying fibrosis severity [14,49,168].

Drugs. Drug-induced steatosis/steatohepatitis is in fact an exclusion criterion for NAFLD, but patients with NAFLD and T2DM/obesity are often pluri-medicated, and there are some suggestions that certain drugs (i.e., acetaminophen, methotrexate, some antibiotics, steroids, tamoxifen, etc.) may elicit more severe (and perhaps more frequent) hepatotoxic effects and favor the progression to NASH/fibrosis in these patients [169,170,171]. The higher hepatotoxic susceptibility of NAFLD patients with diabetes and obesity is probably due to a certain metabolic and inflammatory environment, with hyperglycemia-induced oxidative stress, increased CYP2E1 expression and activity, lower ATP production, increased pro-inflammatory cytokines, mitochondrial dysfunction, etc. [169,172].

On the other hand, multiple drugs have been tested for their potential anti-inflammatory, cytoprotective, and antifibrotic effects in NAFLD patients [3,173,174]. Unfortunately, a great number of them have failed to demonstrate histologic improvement in fibrosis in clinical trials, but a few have shown some promising benefits [174,175,176]. However, there is no drug approved so far for NASH-associated fibrosis in patients with or without T2DM. Details regarding evidence from clinical trials are presented in Table 1 (only trials with positive results with regards to liver fibrosis or ongoing trials with fibrosis-specific endpoints are presented below) [158,177,178,179,180,181,182,183,184,185,186]. For a larger overview of drugs in development for NAFLD-related fibrosis, the reader is referred to references 174–176.

## 5. Liver Fibrogenesis and the Role of Metabolism

The progression of hepatic fibrosis is associated with chronic inflammation, loss of functional hepatocytes, and angiogenesis, but the key element is the over-production and accumulation of extracellular matrix (ECM), which is mainly produced by the activated HSCs that transdifferentiate into myofibroblasts [187,188]. The excessive ECM production seems to be accompanied by a change in its composition with advancing fibrosis, which might result in an increased resistance to degradation and a reduced resolution of fibrosis [187].

The excessive flux of FFAs and carbohydrates to the liver (resulting from exceeding dietary supply and dysfunctional adipose tissue) plays a prime role in the development of liver steatosis, lipotoxicity (generation of lipotoxic species), and subsequent pathogenetic mechanisms, while inflammation is the main driver of fibrogenesis, involving the immune system, vascular system, and soluble mediators [189]. Lipotoxicity results from excessive FFA influx and de novo lipogenesis and leads to mitochondrial dysfunction, oxidative and endoplasmic reticulum stress, apoptosis, and inflammation [173,187]. The hepatocyte lipoapoptosis is in fact a major driver of inflammation, and therefore, it is reasonable to assume that by reducing the metabolic injury, the inflammatory and fibrinogenic responses are attenuated [188]. Apart from the alterations in lipid metabolism, changes in glucose metabolism in the hepatocytes might apparently influence fibrogenesis through the crosstalk with the HSCs, mediated by proteins and perhaps other factors [190]. The hepatocyte stress and apoptosis stimulate inflammatory pathways with recruitment of macrophages and other immune cells, and further production of profibrogenic mediators, such as the TGFβ, which is a potent fibrogenic cytokine that activates HSCs [187]. Other profibrogenic mediators, like Hh ligands, damage-associated molecular patterns (DAMPs), osteopontin, etc., may also activate HSCs [187,188].

The processes of HSC activation and differentiation into myofibroblasts are accompanied by a significant metabolic reprogramming, needed to support these energy-requiring biological functions [187,188,190]. This consists of increased glycolysis, glutaminolysis, lipogenesis, and cholesterol accumulation in HSCs [190]. Some in vitro reports suggest that high concentrations of FFAs, cholesterol, and possibly also glucose may directly activate HSCs and induce the expression of fibrinogenic genes [191,192,193]. During HSC activation, there is a release of lipid droplets containing triglycerides and retinyl esters, an increase in FFAs β-oxidation, and a downregulation of transcription factors like sterol regulatory element-binding protein 1 (SREBP-1c) and PPAR-γ [194,195]. Thus, the pharmacological inhibition of lipogenesis, for example, by metabolic drugs (e.g., inhibitors of acetyl-CoA carboxylase (ACC) or lipoprotein lipase (LPL)) might attenuate fibrosis [190]. Insulin also seems to increase the expression of some fibrogenic signaling molecules, but the role of hyperglycemia and hyperinsulinemia in activating HSCs in the context of NAFLD-associated fibrosis is still not elucidated [162,194]. Hyperglycemia appears rather to amplify fibrogenesis induced by other factors [194]. The metabolic changes also affect the immune cells, mainly the macrophages, which have an important role during hepatic fibrogenesis [190]. Dysregulation of lipid metabolism and trafficking, with accumulation of toxic lipid metabolites, induce the pro-inflammatory phenotype of macrophages/Kupfer cells, while cholesterol crystals stimulate their activation and thus may contribute to fibrosis development [187,196,197,198].

To conclude, systemic and local metabolic derangements are involved in fibrogenic processes. During fibrogenesis, there is also an important metabolic reprogramming in HSCs, hepatocytes, and immune cells, engaging carbohydrate and lipid pathways to support the adaptation to high-energy demands. Preclinical data have indicated that the modulation of cell-intrinsic metabolism and reprogramming of the phenotype of these cells yields beneficial effects on liver fibrosis, but these results need to be substantiated by clinical studies [194].

## 6. Conclusions

Data in the literature point to the fact that between T2DM and NAFLD there is a bidirectional relationship, with one condition influencing the development and progression of the other. T2DM is associated with higher prevalence of NAFLD-associated fibrosis and favors its progression toward more advanced stages, while NAFLD increases the risk of diabetes, mainly in the presence of advanced fibrosis. There are a number of risk factors that impact the natural history of NAFLD, which has a dynamic and variable evolution. The timely recognition and control of the modifiable factors that determine a rapid progression of fibrosis are of critical importance, as the fibrosis stages are positively associated with the risk of liver-related and all-cause mortality.

## Figures and Tables

**Table 1:** Summary of main drugs with potential benefits in terms of NAFLD-related fibrosis (PPAR: peroxisome proliferator activated receptor; RCT: randomized controlled trial; pbo: placebo; NAS: non-alcoholic fatty liver disease activity score; TD: treatment difference; SAF-A: the activity part of the steatosis, activity, fibrosis [SAF] scoring system that incorporates scores for ballooning and inflammation; GLP-1 RA: glucagon-like peptide-1 receptor agonist; GIP: glucose-dependent insulinotropic polypeptide; RR: relative risk; SGLT 2: sodium–glucose cotransporter 2; N/A: not available; wk: week; THR: thyroid hormone receptor; SCD1: stearoyl-CoA desaturase; BID: twice a day; FGF: fibroblast growth factor; HFF: hepatic fat fraction; ELF: enhanced liver fibrosis; [] is presented as 95% confidence interval) [158,177,178,179,180,181,182,183,184,185,186].

Drug Name/ Mechanism of Action	Study/Clinical Trial Ref.	Study Population/Number	Primary Objective	Main Results (If Available)
Monotherapy				
Pioglitazone/PPAR-γ agonist; insulin sensitizer	RCT (pioglitazone 45 mg/day vs. pbo), NCT00994682 [177]	T2DM/prediabetes and biopsy-proven NASH (n = 101)	Reduction of ≥2 NAS points in two histologic categories without worsening of fibrosis.	*Primary outcome*: TD: 41 [23; 59] percentage points; *p* < 0.001. *Additional outcome*: mean change in fibrosis score: TD: −0.5 [−0.9; 0.0]; *p* = 0.039.
Lanifibranor/pan-PPAR agonist	RCT (lanifibranor 1200 mg/800 mg per day or pbo), NCT03008070 [178]	Non-cirrhotic, highly active NASH (n = 247)	Decrease of ≥2 points in SAF-A score without worsening of fibrosis.	*Primary outcome*: RR: 1.7 [1.2; 2.3]; *p* = 0.007 (1200 mg dose vs. pbo); RR: 1.5 [1.0; 2.1]; *p* = 0.07 (800 mg dose vs. pbo). *Additional outcome*: Improvement in fibrosis stage of ≥1 without worsening of NASH: RR: 1.7 [1.2; 2.5] (1200 mg dose vs. pbo); RR: 1.2 [0.7; 1.9] (800 mg dose vs. pbo).
Liraglutide/ GLP-1 RA	RCT (liraglutide 1.8 mg/day vs. pbo), NCT01237119 [158]	Biopsy-proven NASH (n = 52; 17 with T2DM)	Resolution of definite NASH with no worsening of fibrosis.	*Primary outcome*: RR: 4.3 [1.0; 17.7]; *p* = 0.019. *Additional results*: patients with worsening of fibrosis: mean change from baseline vs. pbo: 0.2 [0.1; 1.0]; *p* = 0.04.
Tirzepatide/ Dual GLP-1 and GIP RA	RCT (tirzepatide 5 mg/10 mg/15 mg per wk vs. pbo), NCT04166773 [179]	Biopsy-proven NASH, and stage F2/3 fibrosis, or without T2DM (n = 196 estimated)	Percentage of participants with absence of NASH with no worsening of fibrosis. Secondary outcomes: percentage of participants with ≥1 point decrease in fibrosis stage with no worsening of NASH; percentage of participants with ≥1 point increase in fibrosis stage with no worsening of NASH.	N/A; ongoing; phase 2
Dapagliflozin/ SGLT 2 inhibitors	RCT (dapagliflozin 10 mg/day vs. pbo), NCT03723252 [180]	Biopsy-proven NASH (n = 148 estimated)	Improvement in scored liver histology over 12 months. *Secondary outcome*: change in fibrosis score.	N/A; ongoing; phase 3
Resmetirom/ THR β-selective agonist	RCT (resmetirom (MGL-3196) 80 mg/100 mg per day vs. pbo), NCT03900429 [181]	NASH fibrosis (n = 1759 enrolled)	Proportion with resolution of NASH associated with ≥2-point reduction in NAS without worsening of fibrosis stage *OR* proportion with ≥1-point improvement in fibrosis stage with no worsening of NAS.	N/A; ongoing; phase 3
Aramchol/ partial inhibitor of hepatic SCD1	RCT (aramchol 300 mg BID or pbo), double-blind and open-label; NCT04104321 [182,183]	Biopsy-proven NASH with fibrosis stage F2/3, overweight/obesity, and prediabetes, or T2DM (for double-blind) (150 for open-label; n = 2000 estimated for double-blind)	Proportion of subjects with improvement in liver fibrosis ≥1 and no worsening of NASH. Proportion of subjects with resolution of NASH and no worsening of liver fibrosis.	Open-label interim analysis: Primary outcome: 60.0% had fibrosis improvement of ≥1 stage (of first 20 patients). N/A; phase 3
BFKB8488A/ FGF receptor 1/Klothoβ agonist	RCT (individualized or fixed doses of BFKB8488A vs. pbo), NCT04171765 [184]	Biopsy-proven NASH with stage F2/3 fibrosis, and liver fat ≥8%	Proportion of participants with NASH resolution without worsening of fibrosis. *Secondary outcome*: proportion of participants with improvement in liver fibrosis of ≥1 stage and no worsening of NASH.	N/A; phase 2
Efruxifermin/ Fc-FGF21 fusion protein; FGF receptor agonist	RCT (efruxifermin 28 mg/50 mg/70 mg per wk vs. pbo), NCT03976401 [185]	Biopsy-proven NASH and ≥10% liver fat content (n = 80)	Absolute change from baseline in HFF measured by magnetic resonance imaging. *Secondary outcomes*: change from baseline in liver stiffness; change from baseline in non-invasive biomarkers including liver fibrosis.	*Primary outcome*: absolute changes in HFF: −12.3%, −13.4%, and −14.1% (28, 50, and 70 mg) vs. 0.3% (pbo); *p* < 0.0001. *Additional outcomes*: reduction in ELF scores (*p* = 0.0008 (28 mg), *p* = 0.0005 (50 mg), and *p* = 0.03 (70 mg) vs. pbo); 55% (across all efruxifermin arms with liver biopsies) had a fibrosis improvement of ≥1 stage (no statistical analysis vs. pbo).
Combination therapy				
Dasatinib and Quercetin/tyrosine kinase inhibitor and flavonoid	RCT (dasatinib (100 mg/day) plus quercetin (1000 mg/day) on three consecutive days for three consecutive wks), NCT05506488 [186]	Biopsy-proven NAFLD with stage >F2 fibrosis, but no cirrhosis (n = 30 estimated)	Improvement of fibrosis with at least 1-point without worsening of fibrosis and NAFLD score based on histology.	N/A; ongoing; phase 1, 2

## Data Availability

Data is available upon request.
